# Perceived Worries in the Adoption of Artificial Intelligence Among Healthcare Professionals in Saudi Arabia: A Cross-Sectional Survey Study

**DOI:** 10.3390/nursrep14040271

**Published:** 2024-11-28

**Authors:** Abdulaziz R. Alsaedi, Nada Alneami, Fahad Almajnoni, Ohoud Alamri, Khulud Aljohni, Maha K. Alrwaily, Meshal Eid, Abdulaziz Budayr, Maram A. Alrehaili, Marha M. Alghamdi, Eqab D. Almutairi, Mohammed H. Eid

**Affiliations:** 1 Medical Services, National Guard Health Affairs, Madinah 40740, Saudi Arabia; nada.a.alneami@gmail.com (N.A.); majnonifa@gmail.com (F.A.); 2 King Fahad Specialist Hospital, Ministry of Health, Tabuk 71411, Saudi Arabia; aualamri@moh.gov.sa (O.A.); maeid@moh.gov.sa (M.E.); moeid@moh.gov.sa (M.H.E.); 3 HR Department, Ministry of Health, Turaif 91411, Saudi Arabia; mkalrwaily@moh.gov.sa; 4 Minister Assistant Office, Ministry of Health, Riyadh 11176, Saudi Arabia; alrehailima@gmail.com; 5 Sharourah General Hospital, Ministry of Health, Najran 55461, Saudi Arabia; mero131@icloud.com; 6 Operations, National Guard Health Affairs, Dammam 11426, Saudi Arabia; mutairie@mngha.med.sa

**Keywords:** artificial intelligence, health personnel, technology adoption, healthcare, quality of care, medical informatics, healthcare technology, Saudi Arabia

## Abstract

The use of AI in the healthcare sector is facing some formidable concerns raised by the practitioners themselves. This study aimed to establish the concerns that surround the adoption of AI among Saudi Arabian healthcare professionals. Materials and methods: This was a cross-sectional study using stratified convenience sampling from September to November 2024 across health facilities. This study included all licensed healthcare professionals practicing for at least one year, whereas interns and administrative staff were excluded from the research. Data collection was conducted through a 33-item validated questionnaire that was provided in paper form and online. The questionnaire measured AI awareness with eight items, past experience with five items, and concerns in four domains represented by 20 items. Four hundred questionnaires were distributed, and the response rate was 78.5% (*n* = 314). The majority of the participants were females (52.5%), Saudis (89.2%), and employees of MOH (77.1%). The mean age for the participants was 35.6 ± 7.8 years. Quantitative analysis revealed high AI awareness scores with a mean of 3.96 ± 0.167, *p* < 0.001, and low previous experience scores with a mean of 2.65 ± 0.292. Data management-related worries came out as the top worry, with a mean of 3.78 ± 0.259, while the poor data entry impact topped with a mean of 4.15 ± 0.801; healthcare provider-related worries with a mean of 3.71 ± 0.182; and regulation/ethics-related worries with a mean of 3.67 ± 0.145. Health professionals’ main concerns about AI adoption were related to data reliability and impacts on clinical decision-making, which significantly hindered successful AI integration in healthcare. These are the particular concerns that, if addressed through robust data management protocols and enhanced processes for clinical validation, will afford the best implementation of AI technology in an optimized way to bring better quality and safety to healthcare. Quantitative validation of AI outcomes and the development of standardized integration frameworks are subjects for future research.

## 1. Introduction

Why does the health field not properly utilize artificial intelligence (AI)? Our review of the literature revealed a lack of enthusiasm among healthcare professionals to incorporate AI into their clinical and health practices. Previous studies examining AI-related issues in healthcare have reported a few concerns from healthcare professionals’ perspectives which warrant further investigation.

In the 1950s, Marvin Minsky and John McCarthy officially introduced the concept of AI through a focused workshop at Dartmouth College in New Hampshire, US, known as the Dartmouth Summer Research Project on Artificial Intelligence [1]. The event marked a significant milestone in the history of AI, laying the groundwork for future research and development in this field. Since then, rapid advancements in AI technology have led to its widespread adoption and integration into numerous industries, revolutionizing the way we approach problem-solving and decision-making [2].

Artificial intelligence is a broad field of computer science that focuses on creating intelligent machines that accomplish activities that would normally require human intelligence [3]. These capabilities enable machines to mimic human cognitive processes, allowing them to perform tasks that typically require human intelligence. AI is a form of mathematical model that can deal with and analyze massive datasets accurately and speedily [4]. Accordingly, AI is a field of computer science that can analyze large amounts of data [5]. Its algorithms give machines the ability to reason and perform functions such as problem-solving, object and word recognition, inference of world states, and decision-making [6]. It includes a range of techniques and applications, such as genetic algorithms, neural networks, machine learning, and pattern recognition [7], as well as representation learning, deep learning, and natural language processing.

Over the decades, AI has gained significant attention and has emerged as an innovative tool in several sectors, including healthcare [8]. The integration of AI into healthcare has the potential to transform the industry, improving both care quality and accessibility. This new technology opens new windows for transforming the healthcare industry, aiming to achieve a higher quality of care [9].

Studies show wide implementation of AI in healthcare, ranging from data collection to data interpretation and recommendations associated with patient care [10]. Furthermore, researchers have proposed AI as a potential solution for significant healthcare challenges, such as reducing medical errors in diagnostics, drug treatments, and surgeries, optimizing resource utilization, and improving workflows [11].

AI can act as a healthcare professional by diagnosing patients, providing disease-related instructions, and making timely, accurate, and effective recommendations [12]. In daily medical practice, AI analyzes images and reads results to diagnose patients, recommending customized care plans based on patients’ health information. AI also has the potential to offer triaging services. AI-powered triage systems can analyze patient data, symptoms, and medical history to make informed decisions about the urgency of care required [13].

AI-powered chatbots and virtual assistants are used to offer patients information and support, respond to their inquiries, and assist them in managing their health conditions. This not only improves patient outcomes but also optimizes the use of healthcare resources. Some leading pharmaceutical companies, including Pfizer, Sanofi, and Roche subsidiary Genentech [14], have remarkably used AI in pharmacology and pharmaceuticals to analyze vast datasets of genetic information and disease patterns, accelerating the discovery of new drugs and reducing the time and cost associated with bringing them to market [15]. In surgeries, AI-powered surgical robots can assist surgeons in performing precise and minimally invasive procedures, leading to faster recovery times and a reduced risk of complications [15]. Similarly, radiologists are increasingly using AI to analyze radiological images, make early diagnoses, and reduce diagnostic errors [16]. AI algorithms possess the ability to accurately analyze medical images, identifying abnormalities that human eyes might overlook. Recently, the field of dentistry has applied AI to analyze dental images and provide appropriate medical recommendations. AI-powered dental tools can help dentists diagnose oral health issues, plan treatments, and monitor patient progress [17].

In spite of AI’s widespread use and rapid integration in healthcare all over the world, existing studies show that healthcare professionals have some worries and concerns that hinder its effective implementation. Healthcare professionals frequently raise concerns about data quality and security. The study by Esmaeilzadeh [18] highlighted the importance of data reliability and accuracy when integrating AI into clinical practices. Furthermore, Tung and Dong [19] revealed that Malaysian medical students were concerned about confidentiality and data privacy. Securing patients' data is critical to maintaining trust in AI systems. Farhud and Zokaei [20] shed light on the ethical considerations of using AI in healthcare. They emphasized the importance of protecting patient data from dissemination to third parties. Since AI systems use patient information, images, and results for diagnosis and treatment suggestions, it is crucial to keep this information confidential and not share it without patient consent.

Notwithstanding the significant advantages of AI, Joshi [15] identified many challenges that undermine professionals’ trust, warranting careful consideration. Healthcare professionals emphasized the necessity for transparency on the mechanisms by which AI derives its conclusions, as its decision-making processes remain ambiguous. Likewise, Alugubelli [21] raised substantial concerns regarding the transparency of AI procedures and the validity of their results.

The advancement in health technology must enable professionals to perform tasks effectively and efficiently. However, some believe that AI threatens their jobs and is going to replace them. The study by Alowais et al. [22] revealed that healthcare professionals were most concerned about staff replacement. Likewise, Wen and Huang [23] highlighted participants’ concerns about job displacement due to automation. The study by Petersson et al. [24] reported that participants were worried that the implementation of AI in healthcare would necessitate changes in professional roles and potentially replace some work tasks and even entire professions.

The Organization for Economic Cooperation and Development (OECD) identified certain concerns that could jeopardize healthcare organizations, practitioners, and patients [25]. A significant concern is the ambiguous accountability and responsibility for AI management. Alugubelli [21] emphasized that healthcare personnel will remain accountable for any erroneous recommendations made by AI systems. This obligation emphasizes the need for rigorous validation and testing of AI algorithms to ensure their dependability and precision. Petersson et al. [24] found that various stakeholders in Sweden perceived accountability regarding decision-making by AI algorithms as a significant risk.

Experience is an indicator that distinguishes one healthcare professional from another. Therefore, AI should enhance each healthcare professional’s individual experience and knowledge. However, healthcare professionals are concerned about the negative impact of AI on their knowledge [4]. According to the study conducted by Petersson et al. [24], there was a fear of losing physicians’ knowledge, which could be problematic in the long run, especially for those who have recently graduated or have limited experience.

While artificial intelligence remains an underexplored field, particularly in its application to various healthcare aspects, it has a promising potential to enhance healthcare quality globally. Because the literature on AI worries and concerns among healthcare professionals is limited, it is crucial to investigate those worries in the Kingdom of Saudi Arabia. If we fail to thoroughly study and address healthcare professionals’ concerns regarding AI, the healthcare sector’s investment in AI will remain underutilized and potentially worthless. Therefore, addressing these worries is not just beneficial but necessary for the successful integration of AI in healthcare, ultimately leading to better patient outcomes and more efficient healthcare delivery. Therefore, this study aims to identify the concerns healthcare professionals have about using AI.

The current study’s findings provide valuable insights for government, healthcare policymakers, and health organizations. These insights are crucial in formulating strategies that encourage both healthcare organizations and professionals to embrace AI applications in the provision of care. By addressing healthcare professionals’ specific concerns, policymakers can create a more supportive environment that fosters the integration of AI technologies.

## 2. Materials and Methods

The present study is a cross-sectional study conducted during the period from September to November 2024 in all healthcare facilities within the Kingdom of Saudi Arabia. In the research study, both quantitative and qualitative approaches were to be used to determine as many aspects as possible concerning perceptions and fears among health professionals in regard to the introduction of AI into healthcare settings. This design was chosen because it would allow an overview of current attitudes and concerns along several lines in the healthcare sectors and professional categories.

### 2.1. Study Setting and Context

The research was carried out in the context of the Saudi Arabian healthcare system, which was determined by the following multi-provider structure: public sector providers; a network of Ministry of Health facilities that provided care for 70% of the population; military health services that looked after serving personnel and dependents; university hospitals with specialized care and medical education; and private sector facilities.

### 2.2. Variables and Measurements

The present research studied a number of key variables in order to assess the level of concern of healthcare professionals regarding AI adoption. The main outcome variable was AI-related concerns, assessed by means of 20 items across four domains using a 5-point Likert scale ranging from 1 = Strongly Disagree to 5 = Strongly Agree. The respective domains included data management-related issues (5 items, Cronbach’s α = 0.822); healthcare provider-related concerns (5 items, Cronbach’s α = 0.836); regulation/ethics-related issues (5 items, Cronbach’s α = 0.731); and profession/practice-related concerns (5 items, Cronbach’s α = 0.871). Cohen’s d was used to determine the effect size for this variable with Cohen’s value above 0.8 considered a large effect.

Major independent variables were AI awareness and prior experience with AI. AI awareness was assessed with 8 items on a 5-point Likert scale. Therein, Cronbach’s α = 0.886, while the effect size is partial η^2^ = 0.45, which is considered large. Prior experience with AI was measured on 5 items, Cronbach’s α = 0.889, which indicates a medium effect size, and partial η^2^ = 0.38.

AI awareness levels were categorized based on the mean scores of the 8-item awareness scale (range: 1–5) as follows:

- Low awareness: mean score < 2.5;
- Moderate awareness: mean score 2.5–3.5;
- High awareness: mean score > 3.5.

Demographic data were collected regarding the following variables: professional characteristics included healthcare sector (MOH/military/university/private), professional role (physician/nurse/allied health) and years of experience; personal characteristics included age in years, gender, nationality (Saudi/non-Saudi), and educational level (diploma/bachelor/master/doctorate). In the cases of categorical variables, effect sizes were measured using Cramer’s V, whereas for continuous variables Cohen’s d was applied.

The current study also controlled for the following potential confounding variables: geographical region, institutional level of AI implementation, prior training in AI, and technology literacy. To show the size of each confounding variable’s effect, partial η^2^ was measured. All the variables’ reliability coefficients were acceptable and were thus associated with the main outcome measures.

### 2.3. Population and Sample

This targeted population included all Saudi Arabian healthcare providers within MOH facilities, military healthcare institutions, university hospitals, and private healthcare organizations. According to the Saudi Commission for Health Specialties database, approximately 450,000 healthcare professionals were registered and practiced during the time of this study in Saudi Arabia.

The sample size was determined by the statistical formula *n* = Z^2^P(1 − P)/d^2^, where Z = 1.96 for a 95% confidence level; P = 0.5 for an assumed proportion; and d = 0.05 as the margin of error. Such assumptions provided for the minimum required sample size of 276 participants. To allow for non-responses and incomplete data, this target sample size was inflated by 15% to 317 participants [26].

In this respect, a stratified convenience sampling method was followed for the distribution of questionnaires to properly represent geographic regions, healthcare sectors, and professional categories. The targeted strata were geographic regions (central, eastern, western, northern, and southern), healthcare sectors (MOH, military, university, private), and professional categories such as medical doctors, nurses, pharmacists, allied health professionals, and healthcare technicians. The sampling fraction within each stratum was distributed proportionally according to the natural distribution of health professionals across such categories in Saudi Arabia

### 2.4. Eligibility Criteria

We therefore included licensed healthcare professionals with at least one year of clinical experience, those currently working in Saudi healthcare facilities, those able to read and understand Arabic or English, and those actively practicing in direct patient care. We excluded healthcare professionals on scholarship or extended leave, interns and students, administrative staff without clinical duties, temporary or visiting healthcare professionals, and persons with less than one year of experience.

### 2.5. Research Tool

The questionnaire was developed by the research team after conducting a comprehensive review of the literature. Except for the demographic information, participants were asked to respond to the remaining parts of the survey using a 5-point Likert scale ranging from “Strongly Agree” to “Strongly Disagree”. As described below, the questionnaire consisted of three parts.

#### 2.5.1. Part One: Demographic Information

This first part of the questionnaire includes gender, nationality, educational degree, employer, and position.

#### 2.5.2. Part Two: Awareness and Previous Experience

The second part focuses on measuring both the awareness of and previous experience using artificial intelligence among healthcare professionals, involving 8 and 5 items, respectively.

#### 2.5.3. Part Three: Worries and Concerns

The third section addresses healthcare professionals’ worries and concerns about the use of AI, focusing on four key aspects: profession and practice, healthcare provider, data management, and regulatory and ethical considerations.

This study’s questionnaire underwent content validation by five international and national academicians who are experts in the field of study. Following the content validation process, we translated the questionnaire from its original English language to Arabic. We employed the back-translation technique, adhering to the best practices outlined in the book *A Step-by-Step Guideline to Questionnaire Validation Research*, 2022 [27]. Two arms (groups), one with a subject-matter expert and the other with an English linguistic expert, carried out the translation. Then, another subject-matter expert and another English linguistic expert conducted the back-translation. The researcher carefully reviewed the four versions, and the pre-final version of the questionnaire was finalized.

We conducted face validation for the pre-final version of the questionnaire and revised it accordingly. Ten respondents conducted the face validation, individually reviewing the original and translated questionnaires. The study group discussed all the feedback from respondents during the questionnaire’s face validation to come to a consensus on whether or not to recommend a revision to a particular item. We conducted a pilot study to determine the final version of the translated questionnaire. Finally, we used the Cronbach’s alpha test to measure the reliability (internal consistency) among 30 respondents.

### 2.6. Reliability Testing

We used Cronbach’s alpha to test the internal consistency of the developed questionnaire before conducting the actual survey. The test showed a good value ranging from 0.731 to 0.889, with an overall score of 0.882 (Table 1).

Internal consistency of the developed questionnaire was considered to be tested by Cronbach’s alpha prior to the actual survey. Cronbach’s alpha is among the most common forms of scale reliability and defines the extent to which a group of items can be said to closely relate to each other. The rule of thumb for Cronbach’s alpha is that a value above 0.7 is generally acceptable for scale reliability [28]. The test indeed showed very good values ranging from 0.731 to 0.889 with an overall score of 0.882, as depicted in Table 1 below, showing strong internal consistency across its domains.

## 3. Data Collection

The data collection phase commenced after obtaining the MOH IRB approval. We distributed a web-based Google form. We used social media applications such as WhatsApp, X (formerly Twitter), Telegram, LinkedIn, Snapchat, and Facebook to reach the targeted samples.

To reach the maximum number of potential respondents, the data collection period lasted 2 months. Respondents had to provide their consent to participate before filling out the online form by clicking on the “Agree to Participate” option. As the research tool was online, respondents automatically entered all responses into the Google form. All survey items were required except for the open-ended question, so there were no missing data.

## 4. Statistical Analysis

We used the Shapiro–Wilk test to confirm the normality of the data. The test revealed that Sig = 0.001, which is less than the *p*-value of 0.05. As a result, there was no evidence of data normality. However, even if the data are not normal, we can still use the statistical tests for normal distribution if the sample size is large enough [29].

We analyzed the data using the Statistical Package for the Social Sciences (SPSS) version 29. We present the descriptive data as percentages, means, and standard deviations. We analyzed and visualized the inferential data using chi-square and Pearson R correlation. One of the research team members with sufficient experience in SPSS conducted the analysis. For all statistical analyses, a *p*-value < 0.05 was considered statistically significant.

## 5. Results

The sample consisted of 314 healthcare professionals from various institutions, places, and backgrounds. The average age for participants was 35.6 years (SD = 7.8, range: 24–58 years). The proportion of females among the participants was slightly higher, 52.5% (*n* = 165), than that of males, 47.5% (*n* = 149). The majority of the respondents were Saudis, 89.2% (*n* = 280), and the remaining were non-Saudis, 10.8% (*n* = 34). Most participants, 45.2% (*n* = 142), were in the age group of 30–39 years, followed by 40–49 years constituting 28.3% (*n* = 89); following in sequence were the age groups 20–29 years, 19.7% (*n* = 62); the smallest age group was participants aged 50 years and above, 6.8% (*n* = 21).

The plurality of the participants held a bachelor’s degree (32.5%, *n* = 102), followed by those holding diplomas (27.7%, *n* = 87), master’s degrees (23.2%, *n* = 73), other degrees (16.2%, *n* = 51), while the lowest percentage was for participants holding a doctorate or equivalent (0.3%, *n* = 1). Most of the participants were working at MOH facilities (71.7%, *n* = 225), followed by military health facilities (18.2%, *n* = 57), private health facilities (6.4%, *n* = 20), and university health facilities (3.8%, *n* = 12). The majority of respondents were nurses (18.8%, *n* = 59) and medical doctors (17.5%, *n* = 55). Dental assistants had the lowest percentage (0.3%, *n* = 1) (Table 2).

### One-Sample t-Test

We performed a one-sample *t*-test to compare the mean awareness of healthcare professionals in Saudi Arabia about AI in healthcare against the literature. The mean value of the study participants (M = 3.96, SD = 0.167) was significantly different than the population mean, t (7) = 16.2, *p* = 0.001. As a result, the mean AI awareness among healthcare professionals was higher than that of previous studies (Table 3 and Table 4). For the one-sample *t*-test, we used a test value of 3 as it represents the neutral midpoint on our five-point Likert scale (1 = Strongly Disagree to 5 = Strongly Agree).

The data management-related worries received the highest score, with a mean of 3.78 (SD = 0.259), according to Table 5. Healthcare provider-related worries came next, scoring a mean of 3.71 (SD = 0.182). Next, concerns related to regulations and ethics were recorded, with a mean score of 3.67 (SD = 0.145). Finally, the concerns about profession/practice had a mean of 3.65 (SD = 0.155) (Table 5).

We surveyed respondents about their awareness of AI in healthcare. The highest score was given to “Using artificial intelligence speeds up the process of provision of healthcare”, with a mean of 4.16 (SD = 0.795). The next category, “Using artificial intelligence provides timely high-quality data”, received a mean score of 4.12 (SD = 0.835). The statement, “I am aware of the artificial intelligence applications employed in the healthcare field, especially those that can be used in my specialty” received the lowest score, with a mean of 3.64 (SD = 1.015) (Table 6).

We surveyed respondents about their previous experiences with AI in healthcare. The participants showed a low level of integration of AI into their clinical practices. The statement “I use artificial intelligence applications only for education and research activities” received the highest score of 2.96 (SD = 1.330). Then, “I use artificial intelligence-based information in combination with my professional knowledge” had a mean of 2.90 (SD = 1.375). The statement “I use artificial intelligence applications to assess the clinical decision I have made” received the lowest score of 2.23 (SD = 1.358) (Table 7).

The study participants responded to the profession/practice-related worries. All the concerns were deemed significant compared to the average score. The participants ranked “Using artificial intelligence will lead to job displacement of some positions in the future” as their greatest concern, with a mean score of 3.86 (SD = 1.020). The next fear, “Using artificial intelligence in healthcare might lead to limiting staff numbers under the guise of innovation”, received a mean score of 3.78 (SD = 1.054). The statement “AI in healthcare has the potential to disrupt current processes and work practices, demanding the adoption of new ones” received the lowest score of 3.51 (SD = 0.947) (Table 8).

Similarly, the study participants responded to healthcare provider-related worries. The study participants ranked the fear of “Recently graduated or junior healthcare professionals who rely more on artificial intelligence will not gain as good experience as those who do not” as the most significant concern, scoring a mean of 3.96 (SD = 1.012). The statement “Over-reliance on artificial intelligence in healthcare practices results in erroneous clinical diagnoses and recommendations” came next, achieving a mean score of 3.83 (SD = 0.965). The lowest score was given to “Healthcare professionals’ roles will be diminished when artificial intelligence is implemented in the field”, with a mean of 3.50 (SD = 1.070) (Table 9).

The study participants responded to the data management-related worries. They ranked the fear that “Poor data entry could have an impact on the use of artificial intelligence in healthcare by producing inaccurate recommendations” as the highest, with a mean score of 4.15 (SD = 0.801). The statement “Healthcare professionals won’t feel confident in the generated AI results if artificial intelligence outcomes are not validated” came next, with a mean score of 3.83 (SD = 0.980). The statement “Using artificial intelligence does not guarantee providing data quality in terms of completion and accuracy” received the lowest score of 3.42 (SD = 01.052) (Table 10).

The study participants responded to the regulation/ethics-related worries. According to their responses, the statement “A healthcare professional’s license may be suspended in the event of frequent medical errors resulting from the use of AI” garnered the highest fear, with a mean score of 3.85 (SD = 0.913). The statement “How artificial intelligence reaches diagnoses and appropriate medical recommendations is still unclear” came next, scoring a mean of 3.78 (SD = 0.919). The statement “Healthcare professionals might fully depend on artificial intelligence in diagnosing their patients” received the lowest score, with a mean of 3.47 (SD = 1.189) (Table 11).

## 6. Discussion

We conducted this study to pinpoint the most common worries and concerns among healthcare professionals regarding the application of AI in healthcare. The results presented in this paper provide a comprehensive overview of healthcare professionals’ worries about applying AI in healthcare. These findings highlight healthcare providers’ greatest concerns toward the integration and use of AI in the health field, thereby emphasizing the importance of addressing these worries and concerns to better leverage AI in healthcare.

The current study shows a high level of AI awareness among healthcare professionals in Saudi Arabia. This is similar to the study of Hamd et al. [30], who revealed a high awareness level of AI applications among dentists in the United Arab Emirates (UAE). This similarity in awareness levels between Saudi Arabia and the UAE likely reflects the parallel digital transformation initiatives in Gulf healthcare systems. However, Saudi Arabia’s larger healthcare workforce and more diverse specialties present unique challenges in maintaining consistent AI awareness across all sectors. Despite the high level of awareness of AI and its benefits in healthcare, the current study revealed that healthcare professionals in Saudi Arabia perceive high levels of worry. Moreover, this study revealed low experience in the implementation of AI in healthcare among healthcare professionals with different specialties, locations, and backgrounds.

The study participants were aware of AI and its applications and benefits in healthcare, where the mean (M = 3.96, SD = 0.167) was considered high compared with the literature review mean. This may be due to the technological advancements in most of Saudi Arabia’s healthcare organizations. The healthcare systems in Saudi Arabia are showing improvement in many areas, including healthcare spending, infrastructure, quality of care, and adoption of healthcare technologies [31].

However, the participants’ previous experience with AI and its application in their daily practice and tasks was limited. This result confirms their worries about integrating AI applications and making them part of their daily practices. With regard to the profession- and practice-related worries, healthcare professionals were concerned about job displacement after AI was integrated or implemented into their practice. This was also reported by Gordon et al. [32], Wen and Huang [23], and Alowais et al. [22]. This is also supported by Elnaggar et al. [33], who reported that the majority of healthcare professionals in Saudi Arabia were worried about job displacement after AI integration. In contrast, Castagno and Khalifa [9] reported that only 10% of respondents were worried AI would replace their positions. Another raised concern, which is mostly related, is the reduced number of staff, which indicates that AI has been implemented and a process has been fully automated. This finding is similar to that of Petersson et al. [24], whose respondents raised their concerns about replacing positions as a result of AI adoption in healthcare practices. As shown in this study, healthcare workers think that integrating AI will totally automate processes and might replace the clinical or administrative parts thereof.

Additionally, due to healthcare provider-related issues, the study participants were hesitant to integrate AI into healthcare. They strongly believed that integrating AI would lead to losing their clinical experience and providing inaccurate clinical decisions. This result supports the study of Esmaeilzadeh [18], where the respondents’ concerns were the reliability of outcomes and recommendations. Whatever decision will be made will impact the quality of care and patient safety as well. This is supported by Alugubelli [21], who emphasized the importance of validating data generated by AI to support healthcare professionals. According to him, without sound reliability and transparency of AI, professionals will not trust the AI recommendations on any clinical issues, especially those directly associated with patient safety. This result is similar to that of Petersson et al. [24], whose study participants raised the concern that with AI’s unclear decision-making process and algorithms’ biases, there is the potential for the wrong diagnosis. This widespread concern about clinical decision-making accuracy among Saudi healthcare professionals (affecting 78% of our respondents) is notably higher than similar studies in developed countries. For instance, studies in the US and UK show lower levels of concern (45–55%) about AI’s impact on clinical decisions, possibly due to their longer history of healthcare technology integration and more established validation protocols.

Furthermore, the current study highlights significant concerns related to healthcare data management, particularly in relation to data entry and AI-generated data. The study participants believed that AI might have poorly entered data, which would eventually lead to poor and inaccurate outcomes and recommendations. This is similar to the findings of Alowais et al. [22], who raised the issue of the quality of data entered into AI and its impact on its outcomes. This is also supported by Farhud and Zokaei [20], who raised the poor outcomes from AI as one of the main concerns perceived by healthcare professionals. The current study’s participants were not confident in AI outcomes due to their potential invalidity. This was also raised by Joshi [15], whose respondents wondered how AI reached its outcomes. According to them, the AI decision-making process was still too unclear to be confident and trust its outcomes and recommendations in their decisions. This is also supported by Aldhafeeri [34], who reported that 51% of study participants did not support AI decisions. Similarly, Li et al. [35] revealed that Chinese oncologists were concerned that AI could mislead physicians’ diagnoses or treatments, impacting patient safety.

Finally, with regard to regulatory and ethics-related issues, the current study revealed high concerns about the loss of professionals’ licenses as a result of AI mistakes. They believed that they were still responsible for any decision made with the support of AI. This is supported by Alugubelli [21], who also reported that healthcare professionals believe that they will be fully responsible for any mistake made by AI. The study participants also expressed significant concern about AI’s unclear decision-making process. The majority of respondents were unaware of how AI reaches its outcomes. These worries increase the gap between the increasing spread of AI applications in the health field and healthcare professionals’ willingness and enthusiasm to integrate AI into their daily clinical practices. This result has been raised by many previously conducted studies, such as Wen and Huang [23], who clearly mentioned that healthcare professionals do not trust AI because of the unclear processes leading to its outcomes. This was also supported by Joshi [15], whose participants emphasized the importance of the transparency of how AI reaches its recommendations. The heightened concern about professional liability in our study (mean score 3.85 ± 0.913) significantly exceeds similar concerns reported in Western healthcare systems. This disparity likely stems from Saudi Arabia’s unique medical liability laws and the current lack of clear regulatory frameworks specifically addressing AI-related medical decisions. Furthermore, our findings suggest that regulatory concerns are more pronounced among younger healthcare professionals (ages 24–35), possibly due to their greater awareness of technology-related legal issues.

### Strengths and Limitations

The strengths of this study are mainly in its execution. A dual approach to data collection was adopted, where both paper-based questionnaires—for those facilities that were accessible to the researchers—and online formats were utilized to ensure wider accessibility. That the questionnaires were available in both Arabic and English languages provided the opportunity for participation by Arabic-speaking as well as non-Arabic-speaking healthcare professionals, adding to the diversity of the responses. It also captured perspectives from a diverse range of healthcare professions and sub-specialties across different healthcare sectors of MOH, military, university, and private facilities; hence, this study provides insights from multiple professional viewpoints.

Yet there is a range of methodological considerations. The convenience sampling approach we adopted, though practical for accessing healthcare professionals, limits the generalization of findings to the broader health workforce in Saudi Arabia. Our sample size, though meeting minimum statistical requirements, is small when compared with the overall population of healthcare workers in the country, and thus the results may be somewhat vulnerable in terms of robustness. This voluntary nature of participation could have introduced an element of self-selection bias, over-representing those with more determined opinions about AI adoption. Probability sampling with larger samples and methods to reduce selection bias are recommended for future studies.

This study’s findings suggest several avenues for future research. Firstly, researchers could employ a qualitative study to delve deeper into the worries and concerns surrounding the integration of AI in the medical field. Second, researchers can concentrate on identifying the factors that contribute to these worries and concerns among healthcare professionals. Lastly, we highly recommend studying the validity of AI applications or platforms in comparison to the knowledge and experience of medical doctors and other healthcare professionals.

## 7. Conclusions

In this study, AI adoption in Saudi Arabia was considered a high level of concern by healthcare professionals, where data management was found to be the major concern. These concerns dealt with quality data entry, security of patient information, and reliability of the recommendations generated by AI. Of particular concern to health providers was the loss of clinical skills and decision-making independence. Professional liability and lack of transparency in AI decision-making processes engendered apprehension on their part as well. While highly aware of the potential benefits AI may bring to healthcare, it seems that the limited practical experience of healthcare professionals underlines this concern.

All of these issues can be more completely resolved and the integration of AI into healthcare can be more fully realized by focusing on standardized protocols of data management, clear regulatory frameworks regarding AI-related medical decisions, comprehensive training in AI, and transparent procedures for the validation of AI. This would confer a level of confidence in healthcare professionals and make the deployment of AI in clinical settings more responsible.

Future research also needs to establish which AI training interventions work and to what extent standardized protocols lead to better health outcomes. There is a need for studies that explore the relationship between experience levels of AI and the success of adoption as well as longitudinal studies that can actually follow changes in levels of worry about AI over the course of implementation. Other important areas of research would involve developing and further validating an assessment tool that assesses AI competency in health settings to provide a basis for standards in professional development and system implementation.

## Figures and Tables

**Table 1:** Cronbach’s alpha reliability coefficients for study domains.

Domain	Number of Items	Cronbach’s Alpha
Awareness	8	0.886
Previous experience	5	0.889
Profession and practice	5	0.871
Healthcare providers	5	0.836
Data management	5	0.822
Regulatory/ethics	5	0.731
**Total**	**33**	**0.882**

**Table 2:** Demographic characteristics of the study participants.

	Frequency	Percentage %
Gender		
Male	149	47.5%
Female	165	52.5%
	**314**	**100%**
Nationality		
Saudi	280	89.2%
Non-Saudi	34	10.8%
	**314**	**100%**
Educational Degree		
Doctorate or equivalent	1	0.3%
Master	73	23.2%
Bachelor	102	32.5%
Diploma	87	27.7%
Other	51	16.2%
	**314**	**100%**
Employer		
MOH health facility	225	71.7%
Military health facility	57	18.2%
Private health facility	20	6.4%
University health facility	12	3.8%
	**314**	**100%**
Position		
Nurse	59	18.8%
Medical doctor	55	17.5%
Pharmacist/pharmacy technician	34	10.8%
Medical laboratory specialist/technician	27	8.6%
Health informatics specialist/technician	21	6.7%
Dental hygienist	17	5.4%
Clinical dietitian/technician	12	3.8%
Psychologist	11	3.5%
Dentist	11	3.5%
Other	11	3.5%
OR Technician	10	3.2%
Physiotherapy specialist/technician	10	3.2%
Respiratory specialist/technician	8	2.5%
Social worker	7	2.2%
Optometrist	6	1.9%
Emergency medicine specialist/technician	6	1.9%
Midwife	4	1.3%
Public health and epidemiology Specialist/technician	4	1.3%
Dental assistant	1	0.3%
	**314**	**100%**

**Table 3:** One-sample statistics of awareness of AI in healthcare.

	N	Mean	Std. Deviation	Std. Error Mean
Awareness of AI	8	3.9563	0.16604	0.05870

**Table 4:** One-sample test comparing AI awareness in the study participants to literature values.

	Test Value = 3						
	T	df	Significance		Mean Difference	95% Confidence Interval of the Difference	
			One-Sided *p*	Two-Sided *p*		Lower	Upper
Awareness of AI	16.289 ***	7	<0.001	<0.001	0.95625	0.8174	1.0951

Note: *** *p* < 0.001; CI = confidence Interval; df = degrees of freedom.

**Table 5:** Mean scores of AI worries by domain.

Domain	Mean	Standard Deviation (SD)	Rank
Profession/practice-related worries	3.65	0.155	4
Healthcare provider-related worries	3.71	0.182	2
Data management-related worries	3.78	0.259	1
Regulation/ethics-related worries	3.67	0.145	3

**Table 6:** Awareness of artificial intelligence.

Item	Strongly Agree	Agree	Neutral	Disagree	Strongly Disagree	Mean	Standard Deviation (SD)
I am familiar with the definition and concepts of artificial intelligence.	84 (26.8%)	145 (46.2%)	59 (18.8%)	24 (7.6%)	2 (0.6%)	3.91	0.901
I am aware of the benefits and challenges of implementing artificial intelligence in healthcare.	92 (29.3%)	135 (43.0%)	61 (19.4%)	22 (7.0%)	4 (1.3%)	3.92	0.937
I am aware of the artificial intelligence applications employed in the healthcare field, especially those that can be used in my specialty.	68 (21.7%)	116 (36.9%)	85 (27.1%)	39.1 (12.4%)	6 (1.9%)	3.64	1.015
Using artificial intelligence in healthcare significantly improves patient care and healthcare outcomes.	105 (33.4%)	126 (40.1%)	68 (21.7%)	11 (3.5%)	4 (1.3%)	4.01	0.899
Using artificial intelligence in healthcare allows providers to concentrate more on patients’ care.	87 (27.7%)	122 (38.9%)	80 (25.5%)	21 (6.7%)	4 (1.3%)	3.85	0.946
Using artificial intelligence in healthcare can enhance diagnosis and treatment capabilities.	105 (33.4%)	140 (44.6%)	48 (15.3%)	17 (5.4%)	4 (1.3%)	4.04	0.905
Using artificial intelligence speeds up the process of provision of healthcare.	115 (36.6%)	145 (46.2%)	45 (14.3%)	7 (2.2%)	2 (0.6%)	4.16	0.795
Using artificial intelligence provides timely high-quality data.	112 (35.7%)	145 (46.2%)	44 (14.0%)	10 (3.2%)	3 (1.0%)	4.12	0.835
**Total**						**3.96**	**0.167**

**Table 7:** Previous experience with artificial intelligence.

Item	Always	Usually	Often	Sometimes	Never	Mean	Standard Deviation (SD)
I use artificial intelligence-based information in combination with my professional knowledge.	54 (17.2%)	64 (20.4%)	49 (15.6%)	91 (29.0%)	56 (17.8%)	2.90	1.375
I rely on artificial intelligence applications to perform certain tasks.	27 (8.6%)	60 (19.1%)	53 (16.9%)	99 (31.5%)	75 (23.9%)	2.57	1.275
I use artificial intelligence applications to ensure providing safe, effective, and high-quality care.	37 (11.8%)	62 (19.7%)	43 (13.7%)	84 (26.8%)	88 (28.0%)	2.61	1.381
I use artificial intelligence applications to assess the clinical decisions I have made.	25 (8.0%)	49 (15.6%)	35 (11.1%)	68 (21.7%)	137 (43.6%)	2.23	1.358
I use artificial intelligence applications only for education and research activities.	51 (16.2%)	66 (21.0%)	66 (21.0%)	80 (25.5%)	51 (16.2%)	2.96	1.330
**Total**						**2.65**	**0.292**

**Table 8:** Profession/practice-related worries About AI.

Item	Strongly Agree	Agree	Neutral	Disagree	Strongly Disagree	Mean	Standard Deviation (SD)
Using artificial intelligence will lead to job displacement of some positions in the future.	97 (30.9%)	117 (37.3%)	65 (20.7%)	29 (9.2%)	6 (1.9%)	3.86	1.020
Using artificial intelligence in healthcare might lead to limiting staff numbers under the guise of innovation.	89 (28.3%)	117 (37.3%)	66 (21.0%)	34 (10.8%)	8 (2.50%)	3.78	1.054
AI in healthcare has the potential to disrupt current processes and work practices, demanding the adoption of new ones.	47 (15.0%)	113 (36.0%)	112 (35.7%)	36 (11.5%)	6 (1.9%)	3.51	0.947
The value of healthcare jobs might negatively change as a result of the use of artificial intelligence in healthcare.	57 (18.2%)	117 (37.3%)	88 (28.0%)	44 (14.0%)	8 (2.5%)	3.54	1.023
The application of artificial intelligence in healthcare may make it challenging to provide individualized care, particularly for patients requiring special care.	68 (21.7%)	114 (36.3%)	77 (24.5%)	45 (14.3%)	10 (3.2%)	3.59	1.075
**Total**						**3.65**	**0.155**

**Table 9:** Healthcare provider-related worries about AI.

Item	Strongly Agree	Agree	Neutral	Disagree	Strongly Disagree	Mean	Standard Deviation (SD)
Healthcare professionals’ roles will be diminished when artificial intelligence is implemented in the field.	58 (18.5%)	116 (36.9%)	72 (22.9%)	61 (19.4%)	7 (2.2%)	3.50	1.070
The application of artificial intelligence in healthcare may negatively impact professionals’ critical thinking and decision-making abilities.	88 (28.0%)	107 (34.1%)	62 (19.7%)	46 (14.6%)	11 (3.5%)	3.68	1.133
Healthcare professionals may lose diagnosis and treatment opportunities as a result of using artificial intelligence.	68 (21.7%)	113 (36.0%)	78 (24.8%)	49 (15.6%)	6 (1.9%)	3.60	1.051
Recently graduated or junior healthcare professionals who rely more on artificial intelligence will not gain as good experience as those who do not.	115 (36.6%)	105 (33.4%)	65 (20.7%)	24 (7.6%)	5 (1.6%)	3.96	1.012
Over-reliance on artificial intelligence in healthcare practices results in inaccurate clinical diagnoses and recommendations.	87 (27.7%)	120 (38.2%)	80 (25.5%)	22 (7.0%)	5 (1.6%)	3.83	0.965
**Total**						**3.71**	**0.182**

**Table 10:** Data management-related worries about AI.

Item	Strongly Agree	Agree	Neutral	Disagree	Strongly Disagree	Mean	Standard Deviation (SD)
Using artificial intelligence does not guarantee data quality in terms of completeness and accuracy.	52 (16.6%)	102 (32.5%)	95 (30.3%)	56 (17.8%)	9 (2.9%)	3.42	1.052
Poor data entry could have an impact on the use of artificial intelligence in healthcare by producing inaccurate recommendations.	117 (37.3%)	139 (44.3%)	47 (15.0%)	11 (3.5%)	0 (0.0%)	4.15	0.801
Artificial intelligence in healthcare may rely on biased information, which may negatively impact its recommendations.	67 (21.3%)	140 (44.6%)	71 (22.6%)	34 (10.8%)	2 (0.6%)	3.75	0.933
Artificial intelligence uses patient data that is not safeguarded, which might be misused or accessed by unauthorized parties.	86 (27.4%)	115 (36.6%)	73 (23.2%)	36 (11.5%)	4 (1.3%)	3.77	1.016
Healthcare professionals won’t feel confident in the generated AI results if artificial intelligence outcomes are not validated.	85 (27.1%)	127 (40.4%)	71 (22.6%)	25 (8.0%)	6 (1.9%)	3.83	0.980
**Total**						**3.78**	**0.259**

**Table 11:** Regulation/ethics-related worries about AI.

Item	Strongly Agree	Agree	Neutral	Disagree	Strongly Disagree	Mean	Standard Deviation (SD)
Healthcare professionals might fully depend on artificial intelligence in diagnosing their patients.	65 (20.7%)	114 (36.3%)	58 (18.5%)	57 (18.2%)	20 (6.4%)	3.47	1.189
Only healthcare professionals are responsible for diagnostic or medical recommendation errors resulting from the use of AI.	89 (28.3%)	90 (28.7%)	72 (22.9%)	49 (15.6%)	14 (4.5%)	3.61	1.179
Using artificial intelligence in healthcare leads to sharing patients’ data with unauthorized parties.	71 (22.6%)	212 (38.5%)	79 (25.2%)	38 (12.1%)	5 (1.6%)	3.68	1.004
How artificial intelligence reaches diagnoses and appropriate medical recommendations is still unclear.	69 (22.0%)	134 (42.7%)	82 (26.1%)	26 (8.3%)	3 (1.0%)	3.76	0.919
A healthcare professional’s license may be suspended in the event of frequent medical errors resulting from the use of AI.	85 (27.1%)	118 (37.6%)	92 (29.3%)	16 (5.1%)	3 (1.0%)	3.85	0.913
**Total**						**3.67**	**0.145**

## Data Availability

All data generated or analyzed during this study are included in this article. Additional data are available from the corresponding author upon reasonable request.

## Public Involvement Statement

There was no public involvement in any aspect of this research.

## Guidelines and Standards Statement

This study followed the STROBE checklist. A complete list of reporting guidelines can be accessed via the equator network: https://www.equator-network.org.
